# Vector competence of pre-alpine *Culicoides* (Diptera: Ceratopogonidae) for bluetongue virus serotypes 1, 4 and 8

**DOI:** 10.1186/s13071-018-3050-y

**Published:** 2018-08-13

**Authors:** Anca Ioana Paslaru, Alexander Mathis, Paul Torgerson, Eva Veronesi

**Affiliations:** 1 National Centre for Vector Entomology, Institute of Parasitology, Vetsuisse Faculty, University of Zürich, Zürich, Switzerland; 2 Section of Epidemiology, Vetsuisse Faculty, University of Zürich, Zürich, Switzerland

**Keywords:** Bluetongue virus, *Culicoides*, Vector competence, Dissemination efficiency, Fluctuating temperature

## Abstract

**Background:**

Bluetongue disease, caused by bluetongue virus serotype 8 (BTV-8), appeared for the first time in the northern part of Europe in 2006, and subsequently rapidly spread causing severe economic losses to the farming industry. The implicated vectors of BTV in Europe are *Culicoides* species within the subgenus *Avaritia* (*C. chiopterus*, *C. dewulfi*, *C. obsoletus* and *C. scoticus*). Epidemiological data from Switzerland have shown that BTV, whose spread was eliminated at an early stage by vaccination campaigns, had not been circulating among livestock at higher altitudes where other species dominate the *Culicoides* fauna. In this study, we investigated the extent that *Culicoides* spp. prevailing at higher altitudes (mainly *C. grisescens*) can act as vectors for BTV.

**Methods:**

*Culicoides* were collected at farms in the pre-alpine region (two sites at 1550 m above sea level, masl, referred to as pre-alpine I; one site at 2030 masl, pre-alpine II) and, for comparative purposes, from the Swiss Plateau (one site, 650 masl). They were fed on bovine blood/BTV suspensions (BTV-1, 4 or 8) and incubated for eight days under a fluctuating temperature regime (13–25 °C, mean 19 °C), reflecting a mid-summer warm spell in the pre-alpine region. Susceptibility to BTV transmission was assessed from head homogenates by RT-qPCR and virus isolation.

**Results:**

Overall, 9196 female *Culicoides* were exposed to the three BTV strains through an artificial membrane, with feeding rates of 14–27%. Survival rates of blood-engorged *Culicoides* females at eight days post-infection depended on both virus serotype and altitude of origin. Virus dissemination (C_q_ ≤ the cut-off value as determined by serial virus dilutions) was confirmed only for BTV-1 in *C. scoticus* (dissemination efficiency 22.5%; 9/40) and *C. obsoletus* (5.6%; 1/18) from the Swiss Plateau area. There was no strong evidence of susceptibility to infection for *Culicoides* from the pre-alpine area when fed with all BTV strains (BTV-1, 4 and 8).

**Conclusions:**

This study confirms the susceptibility of *C. scoticus* and *C. obsoletus* to BTV-1 infection, including under cooler temperatures. *Culicoides grisescens*, which is highly abundant at higher altitudes, cannot be considered a potential vector under these temperature conditions.

**Electronic supplementary material:**

The online version of this article (10.1186/s13071-018-3050-y) contains supplementary material, which is available to authorized users.

## Background

Biting midges of the genus *Culicoides* (Diptera, Ceratopogonidae) are responsible for the transmission of bluetongue virus (BTV) and Schmallenberg virus (SBV) [1–4]. BTV (serotype 8) appeared in the northern and western part of Europe for the first time in 2006 [5, 6]; it rapidly spread and caused enormous damage to the farming industry in many affected countries [7, 8]. Intensive vaccination campaigns against BTV-8 were initiated [9–11], but BTV-8 has recently re-emerged in these areas [12]. Other BTV serotypes are circulating in eastern Europe [13] or in the Mediterranean countries (regularly updated maps of Europe showing the areas and the serotypes of circulating BTV are given on the Animal Diseases and Control Measures sections of the European Commission website.

*Culicoides* species within the subgenus *Avaritia* [*Culicoides chiopterus* (Meigen), *Culicoides dewulfi* (Goetghebuer), *Culicoides obsoletus* (Meigen), *Culicoides scoticus* (Downes & Kettle)] have been implicated as the main vectors of BTV in Europe [14–16], as inferred from high abundance and from virus isolation from specimens at places of virus transmission [17–20]. Furthermore, single experimental assays have confirmed the susceptibility of *C. obsoletus* and *C. scoticus* to BTV-8 [21]. Species of the subgenus *Avaritia*, particularly those of the Obsoletus complex (*C. obsoletus* and *C. scoticus*) are also prevalent (up to > 95% of collected *Culicoides*) in Switzerland up to altitudes of around 1100 meters above sea level (masl), whereas *C. chiopterus* and *C. dewulfi* were found up to this altitude but in very small numbers [22–24]. At higher altitudes, however, other species, particularly of the subgenus *Culicoides* [overwhelmingly *Culicoides grisescens* (Edwards)] dominate the *Culicoides* fauna, representing around 50% of the midges at 1300 masl and > 85% at 2000 masl [22, 24, 25].

Epidemiological data from Switzerland have shown that BTV, whose transmission was eliminated at an early stage by vaccination campaigns [9], was not circulating among livestock at higher altitudes. However, investigations of free-ranging ruminants revealed a PCR-positive chamois originating from the Engadin valley at 2000 masl [26]. Furthermore, the highest altitude where a proven transmission of SBV has occurred in Switzerland is Lenzerheide (canton Grisons) at around 1500 masl (Dr C. Nathues, Federal Food Safety and Veterinary Office, Bern, Switzerland, personal communication). Thus, transmission of *Culicoides*-borne viruses at higher altitude appears possible, but it is neither clear which *Culicoides* species are involved nor whether such transmission could regularly occur under average summer conditions or only exceptionally under warm conditions. Ceratopogonidae of higher altitudes have received very little attention so far, and virtually nothing is known about their role in the transmission of BTV [2, 27]. Whereas monitoring activities in Switzerland included altitudes up to 2100 masl [22, 24, 25], the corresponding activities in other European countries were limited to altitudes below 1400 masl: Sicily 1400 m, Bulgaria 1270 m, France 1200 m, Austria 1190 m, central Italy 1184 m, Spain 1006 m [24, 28, 29]. In all cases light-suction trap collections were dominated by midges of the subgenus *Avaritia*. The capability of an arthropod species to act as a vector for a pathogen (vector competence) is related to several intrinsic and extrinsic factors [30, 31]. This is usually investigated with experiments run under various constant temperature regimes. However, it has recently been demonstrated [32, 33] that daily temperature fluctuations can lead to higher infection/transmission rates in insect vectors compared to constant temperatures. This is of great importance for assessing the probability of pathogen transmission in areas with climates that are marginal for this to occur.

With regard to BTV (there is no corresponding data for SBV), experimental work has shown that the minimal temperatures for these viruses to propagate in midges, which is a prerequisite to infect the salivary glands before transmission to a new host occurs, is around 12 °C but slightly varies among the serotypes [34, 35]. For instance, BTV-1 and BTV-9 replicated at a constant temperature of 12 °C, whereas BTV-4 and BTV-8 barely amplified at temperatures below 15 °C [36]. The average temperatures are in this range at higher altitudes in Switzerland (1600 masl) in the summer months July and August (climate normals Davos, www.meteoswiss.admin.ch).

In this study, we investigated whether and to what extent the *Culicoides* that prevail at higher altitudes can act as vectors for BTV. *Culicoides* were collected from pre-alpine area (1550 and 2030 masl) and, for comparative purposes, from the Swiss Plateau area (650 masl). They were orally exposed to three BTV strains (BTV-1, BTV-4 or BTV-8) and incubated under a fluctuating temperature regime (13–25 °C, mean 19 °C), reflecting a mid-summer warm spell in the pre-alpine region (www.meteoswiss.admin.ch). Specimens with fully disseminated virus (i.e. positive heads) were considered potentially competent for BTV transmission since salivary gland barriers have not been described for *Culicoides* species [37].

## Methods

### Study sites and *Culicoides* trapping

**Fig. 1:**
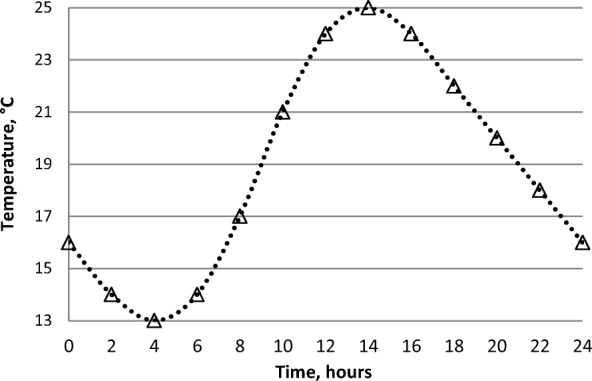
Fluctuating temperature regime during incubation of *Culicoides*

Collections of *Culicoides* biting midges were made at farms in two areas in Switzerland, one in the Swiss Plateau (one site, 650 masl), the other in the pre-alpine region (two sites at around 1550 masl, referred to as pre-alpine I; one site at 2030 masl, referred to as pre-alpine II) (Table 1). Insects were trapped alive as described [38]*.* The light traps were operated from approximately 1–2 h before sunset to 1–2 h after sunrise, between June and October 2015-2017. Wet cotton pads were placed around the cages during transportation to the laboratory where the cages were transferred to incubators (Panasonic MIR-154, Gunma, Japan) with a fluctuating temperature (13–25 °C, mean 19 °C) and a relative humidity of 85–90% (Fig. 1). Cotton wool pads with 10% sucrose solution were supplemented.

**Table 1:** Areas and features of the sites where *Culicoides* were collected

Area	Site (altitude, masl)	Coordinates	Predominant animal species at farm
Swiss Plateau	Adlisberg (650)	47.22298°N, 008.34565°E	Horses
Pre-alpine I	Davos Wolfgang (1575)	46.82638°N, 009.85732°E	Sheep and pigs
	Lenzerheide (1542)	46.73014°N, 009.56091°E	Horses
Pre-alpine II	Davos, Clavadeler Alp (2030)	46.76650°N, 009.81831°E	Cattle

### Insect identification

*Culicoides* were first morphologically classified to group or complex level using an online identification key [39], whereas species identification was achieved on thoraxes by using matrix-assisted laser desorption/ionization time-of-flight mass spectrometry (MALDI-TOF MS) exactly as described [24]. Briefly, thoraxes with wings were manually homogenized in 7 μl of formic acid (25%). One microliter was spotted onto a steel target plate in duplicates, air-dried at room temperature and overlaid with 1 μl of SA Matrix (20 mg sinapic acid in 300 μl acetonitrile, 200 μl distilled water and 1.5 μl trifluoracetic acid; Sigma-Aldrich, Buchs, Switzerland). The plates were sent to Mabritec SA (Riehen, Switzerland) by overnight courier. Protein mass fingerprints were obtained using a MALDI-TOF Mass Spectrometry Axima^TM^ Confidence machine (Shimadzu-Biotech Corp., Kyoto, Japan) and subjected to automated identification against the in-house database containing validated biomarker mass sets of 14 *Culicoides* species. Alternatively, PCR and sequencing were done as follows: DNA from abdomens was isolated by using the DNA mini kit following the manufacturer’s instruction (Qiagen, Hilden, Germany), with proteinase K incubation periods between 4 h and overnight. PCRs targeting the mitochondrial cytochrome *c* oxidase subunit (*cox*1) were done using the the AmpliTaq Gold 360 Master Mix (Applied Biosystems, Thermo Fisher Scientific, Reinach Switzerland) with primers C1-J-1718 and C1-N-2191 mod [40] and, in case of unsuccessful amplifications, with primers LCO1490 and HCO2198 [41]. Each PCR round included the positive and negative control. Amplifications were done in a PTC-200 Peltier Thermal Cycler (Bio-Rad Laboratories AG, Reinach, Basel, Switzerland) with a profile including a Taq DNA polymerase activation step (95 °C for 15 min), 35 cycles at 95 °C for 30 s annealing at 50 °C for 30 s and extension at 72 °C for 60 s. A final elongation step at 72 °C for 10 min was included. DNA concentration was measured using a Nanodrop photometer (NanoDrop products, Wilmington, USA). Sequencing of amplicons after purification with the minelute PCR purification kit (Qiagen), was done by a private company (Synergene GmbH, Schlieren, Switzerland).

### Virus

The three BTV strains used for oral inoculation of *Culicoides* were kindly provided by the Centre for Virus Research (Glasgow, UK): BTV-1 (RSArrrr/01, BSR_1_ passage) [42], BTV-4 (RSArrrr/04, BSR_1_ passage) [43], and BTV-8 (NET06, KC_1_/BHK_3_ passage) [42]. The viruses were further propagated on Vero cells (African green monkey kidney cells), kindly supplied by the Institute of Virology and Immunology (Mittelhäusern, Switzerland) and/or KC cells (*C. sonorensis* embryonal cells), kindly provided by The Pirbright Institute (Pirbright, UK)*.* Briefly, Vero cells were grown in 150 cm^2^ cell culture flasks containing Glasgow Minimum Essential Media (GMEM) (Gibco, Thermo Fisher Scientific, Reinach, Switzerland) supplemented with 1% antibiotics and fungizone (1000 IU/ml penicillin/streptomycin; 4 μg/ml amphotericin; Gibco, Thermo Fisher Scientific) (GMEM complete) and 10% foetal calf serum (FCS, Bioconcept, Allschwil, Switzerland) (GMEM growth media). Flasks were incubated at 37 °C with 5% CO_2_ until reaching a confluence of 75–80% (approximately 24 h). The media was then removed and 200 μl of BTV viral stock was added. After 30 min at room temperature, 40 ml of GMEM maintenance media (GMEM complete supplemented with 2% FCS) were added and the flasks incubated at 37 °C with 5% CO_2_ until the observation of cytopathic effect (CPE) (maximum seven days). Virus titres of supernatants were calculated by titration of 10-fold serial dilutions in 96-well plates as described [44].

KC cells were grown in 75 cm^2^ cell culture flasks containing Schneider’s *Drosophila* Media (Gibco, Thermo Fisher Scientific) supplemented with 1% antibiotics and fungizone (Schneider’s complete media) and 10% FCS (Schneider’s growth media). After seven days of incubation at 27 °C without CO_2_, the supernatant was removed and 100 μl of BTV viral stock was added to the cells, together with 30 ml of Schneider’s complete media with 2% FCS (Schneider’s maintenance media). The flasks were incubated for seven days at 27 °C, and virus infectivity investigated by titration of 10-fold serial dilutions of the supernatants in 96-well plates layered with KC cells according to the protocol described above for Vero cells, but using Schneider’s complete media instead of GMEM. Since KC cells do not show CPE, viral RNA was extracted from the wells after seven days of incubation, and reverse transcription quantitative polymerase chain reaction (RT-qPCR) (see below) was performed to calculate the tissue culture infectious dose (TCID_50/_ml) [45]. Finally, TCID_50/_ml was transformed into PFU/ml, and a standard curve for each BTV strain was generated by conversion into PFU of viral RNA (C_q_ values) determined for each serial dilution.

### Vector competence

#### Culicoides oral exposure to BTV strains

Three days post-collection, the insects were starved for one day and, at day 5, the live ones were transferred, using a mouth aspirator, to a paper cup covered with a net. The insects were anesthetised at -20 °C for 2–3 min and transferred (maximum 300 individuals/chamber) into a “feeding chamber” as previously used for feeding *C. imicola* in South Africa [46], but with Nescofilm50 MMx 40M (Alfresa Pharma Corporation, Osaka, Japan) instead of chicken skin as a membrane (see Additional file 1: Figure S1). The insects were exposed for 30–45 minutes to heparinised bovine blood (obtained from a local abattoir) spiked with virus at a temperature of 25 ± 4 °C. They were then anesthetised as described above and gently transferred into a precooled (-20 °C) glass Petri dish placed on top of an icepack element inside a glove box. The engorged *Culicoides* females were sorted and counted under a stereomicroscope and incubated for eight days in cardboard boxes [Adelphi healthcare packaging 4 oz. (162 ml) A.P.D Deep, Hawards Heath, UK] (maximum 100 females/box) under the fluctuating temperature regime shown in Fig. 1. All *Culicoides* surviving the eight days incubation period were individually stored in sterile 1.5 ml Eppendorf tubes at -80 °C until further investigation. The non-engorged insects were killed and the *Culicoides* specimens counted. One blood-fed female (Day 0) and an aliquot of the blood used for feeding were collected for each meal and strain and stored at -80 °C for further analyses.

A preliminary test evaluating *Culicoides* feeding rates in relation to different ratios of blood to cell culture media (DMEM) was carried out with midges from the Swiss Plateau site at day five post-collection.

Feeding rates of midges on different ratios (1:1, 2:1, 9:1) of bovine blood to virus cell culture media (DMEM) were evaluated with the feeding chamber in triplicate experiments following 3 days of acclimatization and 24 h of starvation.

#### Culicoides dissection and homogenisation

Homogenates of individual heads were used to investigate virus dissemination and thoraxes for species identification (described above). Briefly, heads were removed by using sterile needles (Fine-Ject, Tuttingen, Germany) and transferred into 1.5 ml Eppendorf tubes containing 100 μl of GMEM growth media (see above). For homogenization, *Culicoides* were either manually processed for 30 s using sterilised polypropylene pestles (Sigma-Aldrich, Gillingham, Dorset, UK) mounted to a motorized grinder (Micro Handrührer, Carl Roth, Karlsruhe, Germany) or homogenized for 2 × 30 s with 3 mm diameter stainless steel ball bearings using the Tissue Lyser II Instrument (Qiagen) as previously described [47]. After homogenization, each sample was filled up with 900 μl of Glasgow MEM giving a final volume of 1 ml/homogenate. All tubes were then vortexed and centrifuged for 5 m at 13000× *g*. Aliquots were immediately processed for viral RNA extraction and the rest of the homogenates was kept at 4 °C.

### Virus detection and quantification

#### RNA extraction and PCR

Nucleic acid extraction was carried out using the QIAamp viral RNA mini kit (Qiagen) following the manufacturer’s instructions (elution volume 45 μl). RNA was initially isolated from pools of maximum five individual head homogenates (100 μl each), and viral RNA was extracted from each individual homogenate in case of a positive RT-qPCR on the RNA from the pooled samples. RNA was amplified by RT-qPCR in a CFX96 Touch real-time system (Bio-Rad Laboratories, Cressier, Switzerland). The 25 μl reactions included 5 μl RNA, 0.5 μl of each primer and 1 μl probe (20 μM and 5 μM, respectively), 4.8 μl of RNase-free H_2_O, 12.5 μl of iTaq universal probes reaction mix (2×) (iTaq Universal probes one-step kit, Bio-Rad Laboratories, Hercules, California, USA) and 0.7 μl of iScript advanced reverse transcriptase. Primers [sense BTV_IVI_F (5'-TGG-AYA-AAG-CRA-TGT-CAA-A-3'), anti-sense BTV_IVI_R (5'-ACR-TCA-TCA-CGA-AAG-GCT-TC-3')] and probe BTV_IVI_P (5'-FAM-ARG CTG CAT TCG CAT CGT ACG C-3'-BHQ-1)] were kindly supplied by the Institute of Virology and Immunology (Mittelhäusern, Switzerland). As positive and negative PCR controls, 5 μl of extracted BTV RNA from virus stock or RNase-free H_2_O, respectively, were used. The method used in our tests is an adaption from [48] capable of detecting all BTV serotypes and strains currently circulating and targeting BTV segment 10 (NS3). The reactions were run with the following cycle conditions: 1 cycle for reverse transcription (10 min at 50 °C), 1 cycle for reverse transcriptase inactivation and Taq activation (2 minutes at 95 °C), and 50 amplification cycles (15 s at 95 °C, 30 s at 56 °C, and 30 s at 72 °C).

In order to identify the species that were susceptible for each BTV strain, all the survived females fed with BTV-1, BTV- 4 and BTV-8 that came up positive by RT-qPCR were processed by MALDI-TOF MS or PCR/sequencing. Due to time and mainly budget restriction issues, species identification was not carried out for all the remaining specimens (RT-qPCR negative females). Thus, all the negative females from the BTV-1 experiments were identified, whereas subsamples were analysed from the corresponding BTV-4 and BTV-8 experiments (more than 100 negative females/strain).

#### Virus isolation

All the females that were positive by RT-qPCR were also tested for the presence of infectious virus particles by virus isolation on Vero cells using 25 cm^2^ cell culture flasks following the same protocol described above for virus propagation and amplification. Briefly, 100 μl of head homogenate was inoculated onto the 25 cm^2^ culture flasks layered with 70–80% confluent Vero cells, followed by incubation for 30 min at room temperature. Then GMEM maintenance media was added to a final volume of 8 ml per flask. After incubation for 7 days at 37 °C with 5% CO_2_, a further (blind) passage was carried out by inoculation of 100 μl of the supernatants of passage 1 (V_1_) into new 25 cm^2^ flasks containing Vero cells and amplified as described above, generating passage V_2_. When CPE were observed, titration of the flask supernatant was carried out as described above for viral strains. If no CPE was detected after two passages, no further tests were performed with the sample.

### Statistical analysis

Data on dissemination (positive *Culicoides* heads) for the BTV strains were analysed using a generalized linear model with a Tweedie link to model positive PCR values as a quantitative continuous distribution with an excess of zeros (or negative PCR). The data were also analysed using a binomial link function with positive and negative PCR. The analyses were performed using R (https://www.R-project.org/). A multivariable logistic additive regression model was used to analyse whether altitude of the sampling site or the strain had an (non-linear) impact on the survival rate of blood-fed *Culicoides*. Statistical differences on feeding rate according to dilution factors were investigated using Fisher’s exact test. The significance threshold was set at 0.05.

## Results

### Oral feeding

To evaluate feeding rates, a total of 690 midges collected on a single occasion at the Swiss Plateau site were used. Thus, the feeding rates increased with decreasing blood dilution, with a statistically significant difference between the feeding rates on the blood meals with the 1:1 and 1:9 (Fisher’s exact test, *P* = 0.003) (Table 2). Feeding rates of the vector competence experiments (see Table 3) were done with blood-meals with ratios 1:1 or 2:1 (see below) and were in the range of 14–27% (mean of 18%).

**Table 2:** Feeding rates of Swiss Plateau *Culicoides* after exposure to different ratios of heparinised bovine blood to cell culture medium (DMED) in three replicates (Fisher’s exact test)

Ratio	No. of *Culicoides* exposed to blood/engorged (%)	Mean feeding rate (%)
1:1^a^	69/9 (13)	14
	120/8 (7)	
	80/14 (18)	
2:1^ab^	86/6 (7)	19
	104/26 (25)	
	82/17 (21)	
9:1^b^	84/12 (14)	28
	32/15 (47)	
	33/14 (42)	

*Notes*: *Culicoides* were exposed to blood meal at day 5 post-collection (after incubation for 3 days with access to 10% sucrose and 24 h starvation). The feeding device of [39] with Nescofilm as membrane was used

^ab^*P* = 0.003

**Table 3:** Description of *Culicoides* populations from different areas screened for virus presence in the head (dissemination) after exposure to blood spiked with bluetongue virus (BTV-1, BTV-4 or BTV-8)^a^

Area	BTV strain	No. *Culicoides*: blood-fed/examined for BTV/positive for BTV^b^ (infection rate, %)	Species^c^		
			RT-qPCR positive species (% of all specimens tested)	C_q_ range	RT-qPCR negative species (% all specimens tested)
Swiss Plateau (650 masl)	BTV-1	160/61/27 (44)	24 *C. scoticus* (39)	33.2–41.3	16 *C. scoticus* (26)
			2 *C. obsoletus* (3)	35.7–39.6	16 *C. obsoletus* (26)
			1 *C. pallidicornis* (2)	39.6	2 unknown (3)^d^
	BTV-4	242/104/2 (2)	2 *C. scoticus* (3)	45.4–48.9	46 *C. obsoletus* (75)
					7 unknown (11)
					5 *C. scoticus* (8)
					1 *C. chiopterus* (2)^d^
	BTV-8	407/145/3 (2)	2 *C. obsoletus* (7)	40.2–40.9	20 *C. obsoletus* (67)
			1 *C. pallidicornis* (3)	39.9	6 *C*. *scoticus* (20)
					1 *C*. *punctatu*s (3)^d^
Pre-alpine I`(≈1550 masl)	BTV-1	239/64/1 (1.5)	1 *C. reconditus*-like^e^ (1.5)	40.3	28 *C*. *grisescens* II (44)
					23 *C. obsoletus* (36)
					4 *C. grisescens* I (6)
					4 unknown (6)
					2 *C. scoticus* (3)
					1 *C. pulicaris* (1.5)
					1 *C. deltus* (1.5)
	BTV-4	107/35/0 (0)			nd
	BTV-8	24/18/0 (0)			nd
Pre-alpine II (2030 masl)	BTV-1	2/1/0 (0)			1 *C. grisescens* II (100)
	BTV-4	168/17/0 (0)			nd
	BTV-8	202/103/0 (0)			nd

*Abbreviations*: unknown, species not included in database; nd, not done

^a^Titres (log_10_TCID_50_/ml) of the blood meals were 5.50 (BTV-1) or 6.25 (BTV-4, BTV-8). Engorged females were incubated for eight days under a fluctuating temperature regime (13–25 °C, average 19 °C)

^b^C_q_ values < 50

^c^Species identified by MALDI-TOF MS analyses

^d^All specimens examined for BTV dissemination identified to species level (experiments with BTV-1, collected in September-October) or proportions with randomly selected specimens (biting midges from experiments with BTV-4, collected in July-August, and BTV-8, collected in June)

^e^Identified by PCR/sequencing (96% identity with GenBank entry of *C. reconditus*)

### Virus inocula

The final inoculum of BTV-1 was obtained after three passages on Vero cells (BTV-1 V_3_; titre 5.75 log_10_TCID_50_/ml), while the inoculum of BTV-4 and BTV-8 originated from several consecutive passages through *C. sonorensis* (KC) and Vero cells (BTV-4 KC_3_V_4_, titre 6.75 log_10_TCID_50_ /ml; BTV-8 KC_3_V_6_, 6.75 log_10_TCID_50_/ml). Standard curves of the C_q_ values *versus* the converted PFU values obtained from viral RNA extracted from the serial dilutions of each BTV inoculum (see Additional file 2: Figure S2) showed high correlation coefficients for the virus stocks of all three strains (BTV-1, *R*^2^ = 0.966; BTV-4, *R*^2^ = 0.996; BTV-8, *R*^2^ = 0.999).

### Vector competence

#### Culicoides oral exposure to BTV strains

Overall, 9196 field-collected *Culicoides* were exposed in several experiments to blood spiked with BTV-1 (final titre 5.5 log_10_TCID_50_/ml, ratio blood to virus cell culture supernatant 1:1), BTV-4 or BTV-8 (final titres 6.25 log_10_TCID_50_/ml, ratio 2:1). A total of 4431 exposed midges from the pre-alpine areas I and II yielded 742 (17%) engorged *Culicoides* females out of which 238 (32%) survived the incubation period of eight days and were analysed for the presence of virus (Table 3). The blood-feeding rate (17%; 809/4765) and the survival rate (38%, total 310 specimens) of midges from the Swiss Plateau were similar (Table 3).

**Fig. 2:**
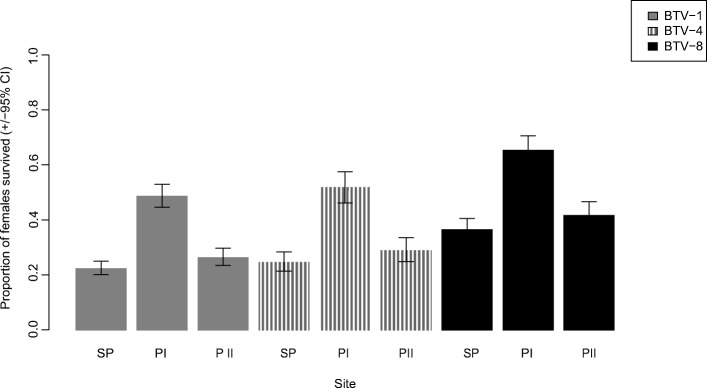
Proportion (± 95% CI) of blood-fed *Culicoides* that survived the 8-day incubation period after exposure to blood spiked with BTV-1, BTV-4 or BTV-8 (Table 4) according to collection site. *Abbreviations*: SP, Swiss Plateau at 650 masl; PI, pre-alpine sites I at 1500 masl; PII, pre-alpine site II at 2030 masl

A multivariable additive logistic regression demonstrated that the survival rate (Fig. 2) was associated with both strain (additive logistic model, *t* = 6.8, *P* = 0.002) and altitude (additive logistic model, *t* = 11.4, *P* = 0.003). *Culicoides* infected with BTV-8 had a better survival rate at each altitude (*P* < 0.0005). The altitude was not a linear effect, i.e. midges from the intermediate site had the highest survival rates with all BTV serotypes.

#### Virus detection and Culicoides species

The C_q_ values of the blood meals used for the vector competence studies and the Day 0 females are shown in Table 4. All Day 0 females were positive for BTV, proving that virus was ingested by fully engorged *Culicoides*.

**Table 4:** Bluetongue viral RNA quantified by RT-qPCR (C_q_) detected in blood used for oral infection of field-collected *Culicoides* and in the abdomen of individual engorged *Culicoides* immediately after feeding (Day 0), and species of analysed specimens identified by MALDI-TOF mass spectrometry using head and thorax

Area	BTV-1			BTV-4			BTV-8		
	Blood	*Culicoides* Day 0	Species	Blood	*Culicoides* Day 0	Species	Blood	*Culicoides* Day 0	Species
Swiss Plateau (650 masl)	24.8	32.8	*C. obsoletus*	26.3	39.0	*C. scoticus*	26.6	35.4	*C. obsoletus*
	27.8	36.4	*C. scoticus*	26.3	43.4	*C. obsoletus*	22.2	38.9	*C. obsoletus*
Pre-alpine I (≈ 1550 masl)	45.1	42.5	*C. pulicaris*	24.4	33.7	*C. grisescens* II	23.2	32.5	*C. grisescens* II
				24.5	37.2	*C. obsoletus*	23.8	37.1	*C. obsoletus*
Pre-alpine II (2030 masl)	28.2	34.4	*C. grisescens* II	25.2	33.7	*C. grisescens* II	23.1	34.5	*C. grisescens* II
				23.0	35.7	*C. grisescens* II			

Overall, 33 *Culicoides* were positive for viral RNA exhibiting C_q_ values ranging between 33.2 and 48.9 (see Additional file 3: Table S1). When applying a cut-off of C_q_ ≤ 36 (BTV-1) and C_q_ ≤ 38 (BTV-4 and BTV-8) as generated from the standard curves of the three BTV strains (see Additional file 2: Figure S2), the number of fully disseminated *Culicoides* is 10 (9 *C. scoticus* and 1 *C. obsoletus*), all of which originated from the Swiss Plateau area and infected with BTV-1. The overall percentage of dissemination efficiency for this population was 16.4% (10/61), represented by 22.5% *C. scoticus* (9/40) and 5.6% *C. obsoletus* (1/18).

The Swiss Plateau *Culicoides* exposed to the three viral strains originated from populations collected during different times of the season (Table 3), and their species compositions varied. Thus, the midges exposed to BTV-1 were collected late in the season (September/October) and mainly contained *C. scoticus* (65.6%; *n* = 40/61) and *C. obsoletus* (29.5%; *n* = 18/61). *Culicoides* tested for BTV-4 (*n* = 104) were collected in July-August. Further analyses of a subsample (*n* = 59/102) from the remaining (negative) *Culicoides* revealed a predominance of *C. obsoletus* (75.4%; *n* = 46), followed by *C. scoticus* (11.4%; *n* = 7). The BTV-8 experiments were done with midges collected in June. Identification of the 3 specimens with detectable viral RNA (although with C_q_ values of 39.9–40.9 which are slightly above the threshold of C_q_ 38) and 27 randomly selected out of the 142 negative ones revealed *C. obsoletus* as predominant species (73.3%; 22/30) followed by *C. scoticus* (20%; 6/30).

Among all the females collected in the pre-alpine areas I and II that were examined for dissemination (viral RNA in the head) of BTV-1, 4 or 8, only one gave detectable viral RNA although with a C_q_ value above the cut-off (C_q_ 40.3) (Table 3). This specimen [as closely related to *Culicoides reconditus* (Campbell & Pelham-Clinton) by barcode sequencing, sequence identity 636/658 bp (96%) with GenBank accession no. KJ767955] originated from the pre-alpine area I and was exposed to blood spiked with BTV-1. The remaining specimens which tested negative for BTV-1 (*n* = 63) mainly belonged to *C. grisescens* (44%) and *C. obsoletus* (36%) (Table 3) as determined by MALDI-TOF MS.

Our study showed that BTV-4 and BTV-8 had lower rates of dissemination compared to BTV-1 (logistic regression, *z*-values = -3.5 and -3.9, respectively, *P* < 0.001 in both cases), and there were no significant differences between BTV-4 and BTV-8.

The quantitative study showed that *Culicoides* infected with BTV-4 and BTV-8 had less viral load compared to those infected with BTV-1 (generalized linear model, *t*-values = -4.1 and -4.6, respectively, *P* < 0.001 in both cases), whilst there were no differences between *Culicoides* infected with BTV-8 and BTV-4.

#### Virus isolation

Overall, homogenates from the heads of the 33 females with detectable viral RNA after RT-qPCR (see Additional file 3: Table S1) were screened for infectious virus particles based on a Vero cells assay. Only one was positive as observed by a cytopathic effect (CPE) after two blind passages. The female that was found positive for virus isolation was a *C. scoticus* (C_q_ 34.8) from the Swiss Plateau which had fed on blood spiked with BTV-1. The virus titre of this female reached 6.5 log_10_TCID_50_/ml after two consecutive amplifications on Vero cells (V_2_).

## Discussion

In this study we describe vector competence traits for field-collected populations of *Culicoides* from pre-alpine and Swiss Plateau areas after oral exposure to three bluetongue virus strains (BTV-1, BTV-4 or BTV-8). Such experimental work has been performed by other research groups, focussing mainly on field-collected *C. imicola* which is the incriminated main BTV vector in Africa [34, 49–51]. Further such studies have also been done in *C. brevitarsis* and *C. insignis* in the USA and in Australia [52, 53] whereas in Europe, *C. impunctatus*, *C. obsoletus*, *C. pulicaris* and *C. scoticus* have been proven to be susceptible to BTV serotypes 3, 4, 8 or 9 [21, 54, 55]. Other studies were also done on laboratory colonies of *C. nubeculosus* and *C. sonorensis* [54, 56–60]. Due to the huge variations in the experimental settings (*Culicoides* species, BTV strain, virus titre in blood meal, incubation temperature and period, etc.), the results are difficult to compare and do not allow general conclusions to be drawn [27].

The vector competence studies here presented were done under fluctuating temperature ranging between 13 and 25 °C (mean of 19 °C), representing a warm summer spell at the pre-alpine region. These are considerably cooler conditions than those in comparable experiments when incubation temperatures were at 23 or 25 °C [21, 54, 55]. Our results show that under these conditions *Culicoides* from Swiss Plateau are susceptible to BTV-1 strain infection (Table 3).

Most striking is the high overall susceptibility (*Culicoides* with viral RNA C_q_ ≤ 36) of the midges from the Swiss Plateau to BTV-1 (16.4%). The most abundant species in these populations, *C. scoticus* (65.6%), had a dissemination efficiency (positive heads/all *C. scoticus* tested) of 22.5% (9/40) (Table 3). High vector competence for BTV-1 has been described before for *C. bolitinos* (26.5%) and *C. sonorensis* (12–20%) whereas *C. imicola* and *C. brevitarsis* seem to be relatively refractory [49, 50, 52, 56].

The susceptibility to fully disseminated infection in our study varied among the BTV strains that we tested. However, it is also true that the number of survived *Culicoides* post-oral-feeding (e.g. BTV-4 infected *Culicoides* from the pre-alpine areas I and II) was small despite the fact that we had a considerable amount of fully engorged midges (Table 3). Although we were aware of this, the short lenght of summer season at pre-alpine altitudes inevitably affected the available period for field *Culicoides* trapping (July-August). This was one of our major constrains during our two-year funded project. Further studies should therefore aim to improve survival rate of blood fed *Culicoides* in order to confirm the lack of susceptibility we here observed for those *Culicoides* from pre-alpine areas (Table 3).

The higher susceptibility observed in BTV-1 might, on the one hand, be due to its adaptation to cooler temperatures. The degree and speed of replication varied for several strains of BTV investigated on a KC cells based assay (*C. sonorensis*) when infected cells were incubated at different temperatures (8, 10, 12, 15, 25, 30 °C) [36]. Indeed, BTV-1 was the only one showing a good amplification rate at temperatures as low as 12 °C, while there was little indication of an increase in virus titre for BTV-4 at 21 days post-infection. Both strains comparably replicated at 25 °C, reaching a plateau of 6.5 log_10_TCID_50_ /ml after only four days.

On the other hand, discrepancies of dissemination observed in our studies should likewise consider the *Culicoides* species composition in the assays. Thus, the experiments with BTV-1 with populations from the Swiss Plateau contained mainly the highly susceptible *C. scoticus* as shown in the present study. The corresponding experiments with midges from the pre-alpine I area, which revealed only one insect with detectable viral RNA (*C. reconditus*-like; C_q_ 40.3), mainly comprised *C. grisescens* (cryptic species I and II, [61]) which seems to be refractory to the virus under the chosen conditions (incubation temperature and time).

Dissemination efficiency of the Swiss Plateau midges was much lower for BTV-4 and BTV-8 as compared to BTV-1, and the C_q_ ranges were just (BTV-8) or clearly (BTV-4) above the cut-off value. As indicated above, the higher temperature requirements of these strains [36] might have contributed to this outcome. Again, the C_q_ values in these experiments were high (see also below), and thus these interpretations need to be taken with extreme caution. Earlier experimental work revealed a similar low vector competence for BTV-8 of less than 1% for both species after incubation at 23–25 °C for ten days [21].

There were strong variations in the population ratios of *C. scoticus* and *C. obsoletus* over the season in our experiments, and such seasonal variations have been described before [62]. As *C. scoticus* and *C. obsoletus* differ in their vector competence for BTV-1 their identification to species level, rather than referring to them as Obsoletus complex, seems essential in vector competence studies. This can be achieved by PCR/sequencing, multiplex PCRs [63], or by mass spectrometry as in the present study.

*Culicoides pallidicornis* was a rare species in the collections from the Swiss Plateau (2 of 152 specimens identified by mass spectrometry). Both specimens had detectable viral RNA for BTV (1 or 8), though with C_q_ values slightly above the threshold (39.6 and 39.9, Table 3). The species was also rare in a study using Onderstepoort light traps in the Netherlands, with a peak activity in early summer [64]. However, in a study from Germany in which midges were directly aspirated from animals (cattle, sheep) or collected in drop traps baited with these animals, *C. pallidicornis* was the second most abundant taxon after the Obsoletus complex (*C. obsoletus*, *C. scoticus*) [65]. The species was also identified as feeding on housed cattle [66]. Using light traps thus seems to largely underestimate the abundance of *C. pallidicornis*, as has been described for other species [67, 68]. Thus, *C. pallidicornis* might play a role in BTV transmission at sites with high abundances, and further studies on the biology and the vector role of this species are required.

Overall, vector competence is only one of the several factors contributing to the vector capacity [69]. Others include abundance and biting rate. Thus, for example, although some species may have high competence rates, they do not serve as good vectors in the field if their abundance (*C. bolitinos* at lower altitudes in sub-Saharan Africa) [70] or biting rate (*C. brevitarsis*) [71] is low. On the other hand, a species with a low competence rate but high abundance (*C. imicola* and *C. fulvus* [70]; Obsoletus group [21]) represents a risk for BTV transmission. Therefore, in our studies, the high abundance of *C. obsoletus* and *C. scoticus* at lower altitudes suggests a considerable vector capacity for these species when infected with BTV-1 at lower temperatures (mean of 19 °C), whereas *C. grisescens*, highly abundant at higher altitudes, cannot be considered a potential vector when infected with same BTV strain.

An explanation for the high C_q_ values recorded in our experiments could be that only the heads were used to determine the viral dissemination but not the thoraxes containing the salivary glands as in other such studies [56, 72]. Another reason might be the incubation temperature (mean of 19 °C) and the relatively short incubation time of eight days. The latter was chosen because preliminary experiments with field-collected *Culicoides* had resulted in high mortality rates afterwards. However, an incubation time of eight days is in line with some previous work on *Culicoides* vector competence [21, 55, 58, 73]. In addition, a previous study [36] on BTV dissemination in laboratory reared *C. sonorensis* incubated at 15 °C showed positive *Culicoides* at day 5 post-infection (C_q_ 32), while no additional positive *Culicoides* were found from day 6 to day 30 post-infection.

Very few studies have been carried out assessing vector competence among field-collected *Culicoides* [27] and this is mainly due to the evidence that European *Culicoides* species are reluctant to artificially feed through membranes under laboratory conditions [70, 74]. As an alternative to membrane feeding, the use of cotton wool soaked with infectious blood has been evaluated. Although good feeding rates were achieved, the infection rates were significantly lower, due to the fact that smaller amounts of blood were engorged [75]. We evaluated different combinations of membranes, devices and attractants [46, 76, 77] (own data not shown), and eventually choose to use the feeding device previously described [39] but with Nescofilm50 MMx 40M as a membrane instead of chicken skin. Thus, feeding rates among field-collected *Culicoides* up to 52% were observed during the evaluation experiments; they varied between 14 and 27% in the vector competence experiments shown in this paper. Variations in feeding rates among populations of field-collected midges have to be expected as the individuals of these populations are of different age and gonotrophic status which may have implications on their predisposition to probe.

According to previous work on the Palaearctic species *C. impunctatus* [74], the length of storage before exposure to blood seems to crucially influence the feeding rates. Indeed, our own preliminary work (not shown) revealed that feeding the midges at days 2, 3 or 4 post-collection (including 24 h of starvation) yielded lower feeding rates (11%, 0% and 5%, respectively) compared to feeding at day 5 as mentioned above.

Several studies described 37 °C as the optimal temperature for the infectious blood during artificial feeding of *Culicoides* [46, 76, 78]*.* In our experience, field-collected *Culicoides* exposed to a blood meal at this temperature barely took blood, and the mortality rate was increased. We therefore opted for lower temperatures of 25 ± 4 °C.

As shown in this paper, feeding rates were also higher when using less diluted blood meals (blood to virus-containing cell supernatant of 9:1, Table 2). However, we could not apply this ratio in our experiments as this would have excessively reduced the infectivity. Earlier studies have suggested minimal virus titres in the blood meals (C_q_ values below 30 per μl blood or a minimum of 5 log_10_TCID_50_/ml) [49, 79]. Thus, 2:1 (BTV-4 and BTV-8) or 1:1 (BTV-1) ratios were used in our experiments to fulfil these requirements.

Although we are aware that when working with field *Culicoides* there are several parameters that can influence their survival rate (e.g. age, stress and dehydration during transportation, and other logistics), we would here like to describe interesting aspects that we observed during our studies.

First, the *Culicoides* populations fed on BTV-1 and BTV-4 had lower survival rates (Fig. 2) comparing to those fed on BTV-8, no matter the altitude from which they originated. We are not aware of any report on such virus-specific differences in survival rates of biting midges. Whereas a meta-analysis of studies revealed that arboviruses reduce the survival of their mosquito vectors [80], nothing is known in this regard to *Culicoides*. Survival rates of these vectors have been evaluated with two pathogens. Whereas the protozoon *Haemoproteus* induced high mortality in *C. impunctatus* [81], no effect was observed in *C. imicola* infected with a bacterium [82]. An unrelated effect of BTV infection was reported by McDermott et al. [83]. The eyes of *C. sonorensis* infected with BTV-17 were affected, leading to a change in field behaviour (but no information is given about survivorship).

Secondly, the *Culicoides* populations from the pre-alpine I area were the most robust, having a higher surviving rate no matter the virus strain used for their infection, while the *Culicoides* from the Swiss Plateau and the pre-alpine II areas had similar survival rates (Fig. 2). The reasons for this are obscure. A possible cause, a seasonal effect, can be ruled out as the midges from the two pre-alpine areas were collected during the same period (July-August) (and the midges from the Swiss Plateau area between June and October). The species compositions at the areas also cannot explain the differences: at the site with the highest surviving rate (pre-alpine I; Table 3) specimens of species belonging to the subgenus *Avaritia* (Obsoletus complex) and the subgenus *Culicoides* (mainly *C. grisescens*) were present in nearly equal numbers. At the Swiss Plateau site, the species of the Obsoletus complex dominated (Table 3), whereas at the pre-alpine II site there were only species of the subgenus *Culicoides*, mainly *C. grisescens* (data from the population dynamics study, to be published elsewhere). Thus, the species composition at the pre-alpine I area, where the midges had the highest survival rates, was in-between to the other populations.

## Conclusions

To our knowledge, this is the first paper investigating the role of *Culicoides* for bluetongue virus transmission at high altitudes. We strongly confirm previous assumptions that *C. scoticus* is a highly suitable vector for BTV in Europe under cooler conditions. Our results have shown that in the pre-alpine regions, where *C. scoticus* is almost absent, there is no strong evidence to support viral dissemination efficiency of BTV. However, midges in these regions might be vectors of other pathogens: the highest known areas where seroconversion to Schmallenberg virus occurred were at altitudes above 1500 masl [84].

## Additional files

Additional file 1: **Figure S1.** *Culicoides* feeding device. *Culicoides* are anesthetised at -20 °C for 2–3 min and transferred moved into a “feeding chamber” (maximum 300 individuals/chamber) with Nescofilm50 MMx 40M (Alfresa Pharma Corporation, Osaka, Japan) membrane (a). The chamber is placed in a plastic cup containing heparinised bovine blood mixed with virus and a stirring magnet (b). The cup is covered with a lid (c) and placed in a glass bowl with (d) and placed on a heating magnetic stirrer (e). The insects were exposed for 30–45 min to a temperature of 25 ± 4 °C. (PDF 106 kb)

Additional file 2: **Figure S2.** Standard curves of all BTV strains used for oral infection of *Culicoides*. Conversion of viral RNA (C_q_ values) into PFU was determined for each serial dilution by RT-qPCR. (PDF 90 kb)

Additional file 3: **Table S1.** Viral RNA quantification cycles (C_q_) recorded from field collected *Culicoides* artificially fed with BTV-1, 4 and 8 spiked blood. Fully engorged *Culicoides* were incubated for 8 days in a climatic chamber under a fluctuating temperature regime (individuals with negative C_q_ values are here not given). Their associated species name was confirmed by morphological features and by matrix-assisted laser desorption/ionization time-of-flight mass spectrometry (MALDI-TOF MS) or barcode sequencing. (PDF 20 kb)

## Abbreviations

BTV: Bluetongue virus
C_q_: Quantification cycle
CPE: Cytopathic effect
DMEM: Dulbecco’s modified Eagle’s medium
FCS: Foetal calf serum
GMEM: Glasgow minimum essential medium
KC cells: *Culicoides sonorensis* embryonal cells
MALDI TOF MS: Matrix-assisted laser desorption/ionization time of flight mass spectrometry
masl: Meters above sea level
PFU: Plaque forming unit
RT-qPCR: Reverse transcription quantitative polymerase chain reaction
SBV: Schmallenberg virus
Vero cells: African green monkey kidney cells
