# Supplementary figures and images for: Vector competence of pre-alpine Culicoides (Diptera: Ceratopogonidae) for bluetongue virus serotypes 1, 4 and 8
Source: Parasit Vectors. 2018 Aug 13;11:466. doi: 10.1186/s13071-018-3050-y (PMC6090685; doi:10.1186/s13071-018-3050-y)

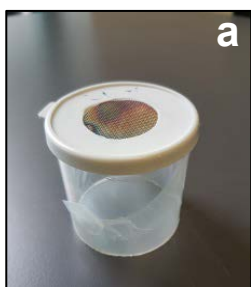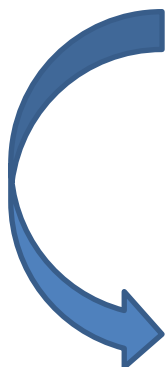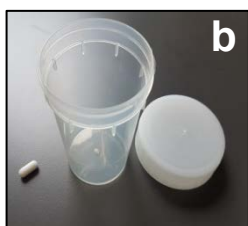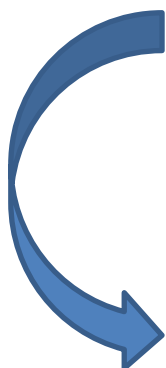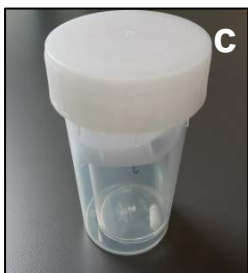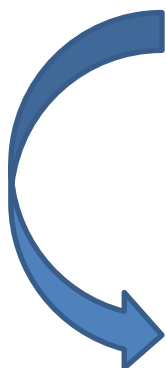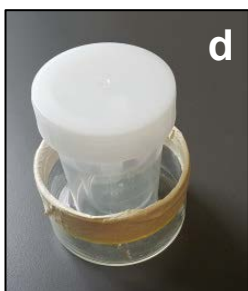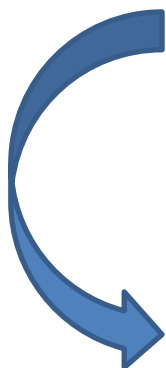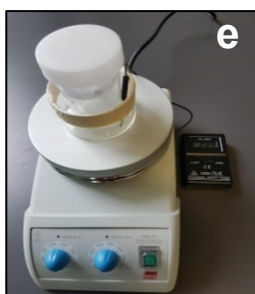

Supplement: Supplementary file 1 — Figure S1. Culicoides feeding device. Culicoides are anesthetised at -20 °C for 2–3 min and transferred moved into a “feeding chamber” (maximum 300 individuals/chamber) with Nescofilm50 MMx 40M (Alfresa Pharma Corporation, Osaka, Japan) membrane (a). The chamber is placed in a plastic cup containing heparinised bovine blood mixed with virus and a stirring magnet (b). The cup is covered with a lid (c) and placed in a glass bowl with (d) and placed on a heating magnetic stirrer (e). The insects were exposed for 30–45 min to a temperature of 25 ± 4 °C. (PDF 106 kb) [file 13071_2018_3050_MOESM1_ESM.pdf]

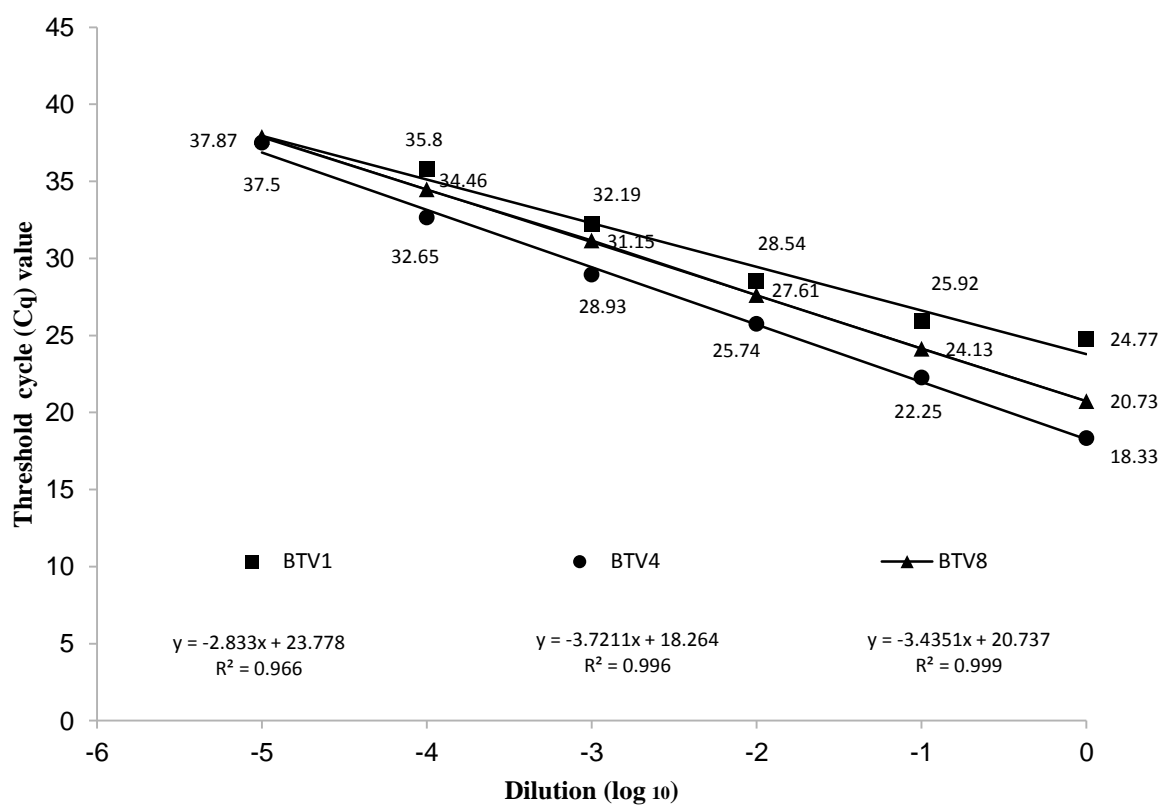

Supplement: Supplementary file 2 — Figure S2. Standard curves of all BTV strains used for oral infection of Culicoides. Conversion of viral RNA (Cq values) into PFU was determined for each serial dilution by RT-qPCR. (PDF 90 kb) [file 13071_2018_3050_MOESM2_ESM.pdf]
