# Supplementary material for: Vector competence of pre-alpine Culicoides (Diptera: Ceratopogonidae) for bluetongue virus serotypes 1, 4 and 8
Source: Parasit Vectors. 2018 Aug 13;11:466. doi: 10.1186/s13071-018-3050-y (PMC6090685; doi:10.1186/s13071-018-3050-y)
Supplement: Supplementary file 3 — Table S1. Viral RNA quantification cycles (Cq) recorded from field collected Culicoides artificially fed with BTV-1, 4 and 8 spiked blood. Fully engorged Culicoides were incubated for 8 days in a climatic chamber under a fluctuating temperature regime (individuals with negative Cq values are here not given). Their associated species name was confirmed by morphological features and by matrix-assisted laser desorption/ionization time-of-flight mass spectrometry (MALDI-TOF MS) or barcode sequencing. (PDF 20 kb) [file 13071_2018_3050_MOESM3_ESM.pdf]

BTV Strains	Species	C_q values
**BTV-1**	*C. scoticus*	33.2<br>34.8<br>35.4<br>35.4<br>35.5<br>36.5<br>36.6<br>36.6<br>36.8<br>37.0<br>37.1<br>37.2<br>37.2<br>38.1<br>38.6<br>38.6<br>38.7<br>39.1<br>39.4<br>40.1<br>40.6<br>40.6<br>40.8<br>41.0
	C. obsoletus	35.7<br>39.6
	C. pallidicornis	39.6
	C. reconditus - like	40.2
**BTV-4**	*C. scoticus*	45.4
	C. scoticus	48.9
**BTV-8**	*C. pallidicornis*	39.9
	C. obsoletus	40.2
	C. obsoletus	40.9
